# Mechanisms underpinning the association between physical activity and mental health in adolescence: a 6-year study

**DOI:** 10.1186/s12966-020-0911-5

**Published:** 2020-01-31

**Authors:** Isabelle Doré, Benjamin Sylvester, Catherine Sabiston, Marie-Pierre Sylvestre, Jennifer O’Loughlin, Jennifer Brunet, Mathieu Bélanger

**Affiliations:** 1 School of Kinesiology and Physical Activity Sciences, Faculty of Medicine, Université de Montréal, Montréal, Québec Canada; 2 CHUM Research Centre, Montréal, Québec Canada; 3 Department of National Defense, Ottawa, ON Canada; 4 University of Toronto, Toronto, ON Canada; 5 Université de Montréal, Montréal, Québec Canada; 6 University of Ottawa, Ottawa, ON Canada; 7 Université de Sherbrooke, Moncton, New Brunswick Canada; 8 Centre de formation médicale du Nouveau-Brunswick, Moncton, New Brunswick Canada; 9 Research Services, Vitalité Health Network, Moncton, New Brunswick Canada

**Keywords:** Physical activity, Autonomy, Competence, Relatedness, Well-being, Basic psychological needs

## Abstract

**Background:**

Physical activity (PA) can promote mental health, but the mechanisms underpinning this association are not well-established. This study examined if perceptions of three basic psychological needs (autonomy, competence, relatedness) and moderate-to-vigorous physical activity (MVPA) mediate the association between number of years participating in PA and mental health in adolescence.

**Methods:**

Participants included 937 children (55% female) age 10–11 at inception of the longitudinal MATCH study, who provided data every 4 months over 6 years. Mediation analyses were used to assess the natural direct effect of number of years of PA participation (cycles 1–15) during late childhood and adolescence on later mental health (cycle 16), measured with the Mental Health Continuum-Short Form (MHC-SF), and the natural indirect effect through each of self-perceived autonomy, competence and relatedness, and self-report MVPA (cycle 15).

**Results:**

In single mediator models, indirect effects of autonomy, competence, relatedness and self-report MVPA were statistically significant. In joint mediation models (each of three models including one basic psychological need and MVPA), autonomy, competence and relatedness mediated 71, 27, and 51% of the association respectively; MVPA mediated 27–31% of the association. In the mediation model including all four mediators, relatedness mediated the largest proportion of the association, followed by autonomy and MVPA.

**Conclusion:**

Results support developing strategies to encourage adolescents to engage and remain involved in PA. This could foster perceptions of autonomy, competence, and relatedness as well as MVPA, which in turn may enhance mental health.

## Introduction

Participation in physical activity (PA) and sport in youth has numerous physical and mental health benefits [1, 2]. It contributes to positive youth development [1, 3] by providing opportunities to develop personal and social skills and by reinforcing self-esteem [4], self-efficacy [5] and self-control [6]. PA and sport provide a context for social integration by allowing participants to be socially connected and accepted as important by others, which can positively impact mental health [7–9] especially when undertaken in a group or team setting [10]. Most evidence to date on the benefits of PA focuses on mental illness rather than mental health, which is a positive psychological construct. Although mental health is inversely related to the risk of anxiety and depression, it is more than the absence of mental illness [11]. Mental health is a multidimensional construct comprising emotional, psychological and social dimensions of well-being [12]. Identifying mechanisms through which PA increase well-being among adolescents is critical to developing effective targeted interventions to promote mental health and reduce mental disorders in youth.

Basic psychological needs [13], defined as organismic necessities essential for psychological growth, integrity and wellness, represent psychosocial mechanisms by which the social environment impacts functioning and well-being [13]. Satisfaction of the three main psychological needs of *autonomy* (feelings of volition and self-governing one’s own behaviour, choices and decisions) [14], *competence* (sense of mastery through effective interaction with the social environment and experiencing opportunities in which to express one’s capabilities) [15], and *relatedness* (feeling a secure sense of belongingness and connectedness to others in one’s social environment) [14] in social contexts predicts health and well-being outcomes [16, 17]. In PA contexts, satisfaction of these needs is associated with enhanced engagement, performance and well-being in adolescents [18, 19]. For example, satisfaction of basic psychological needs predicted increases in well-being in young female gymnasts in the US [20], and in elite adolescent athletes in Norway [21]. In a longitudinal study of adolescent male soccer players, Balaguer et al. [22] found that improvements in satisfaction of basic psychological needs positively predicted changes in subjective vitality and was negatively related to burnout. When PA occurs within a cooperative structure, it can foster connections with others and feelings of volitional engagement [23], and it offers opportunities for enhanced competence if individuals within the group work together [24], provide feedback, support each other’s successes and build friendships [25].

Youth consistently involved in PA during adolescence also report higher levels of self-report moderate-to-vigorous physical activity (MVPA) [26], and convincing evidence indicates increased benefits on mental health with higher self-report MVPA levels [27, 28]. Consistent involvement in PA during chilhood and adolescence might impact mental health by increasing MVPA levels and/or by satisfying basic psychological needs. Thus, MVPA could represent another mechanism underpinning the benefit of PA on mental health.

**Fig. 1:**
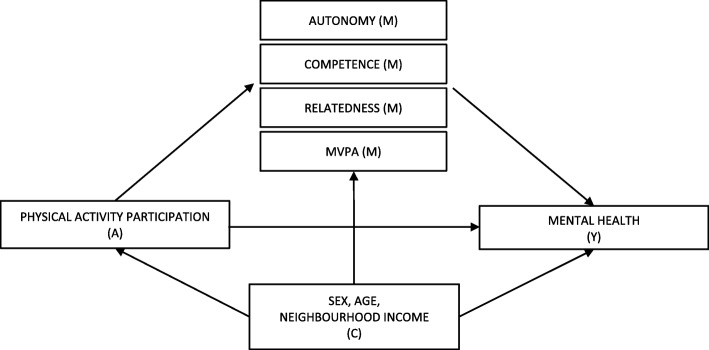
Directed Acyclic Graph representing the potential mediating effect of perceived autonomy, competence and relatedness specific to a PA context and MVPA, on the association between the number of years of PA participation and mental health

Using self-report measures, the first objective of this study was to investigate if perceptions of autonomy, competence, relatedness and/or MVPA in adolescence mediate the association between PA in childhood and early adolescence and mental health in later adolescence (see Directed Acyclic Graph (Fig. 1)). Second, in joint models including two mediators we examined whether perceptions of each of autonomy, competence and relatedness, in addition to MVPA, mediate the association between PA during childhood and early adolescence and mental health in later adolescence. Finally, in a 4-mediator model we examined the specific contribution of each potential mechanism. We hypothesized a direct effect of PA on mental health (i.e., more years of PA is associated with better mental health in later adolescence). We also hypothesized that more years of PA is associated with enhanced perceptions of autonomy, competence and relatedness and higher levels of MVPA, which in turn, are associated with better mental health. We expected that perceptions of autonomy, competence and relatedness and MVPA explain part of the association. Results of this study could inform the development of PA interventions that promote mental health among adolescents.

## Methods

### Study population and procedures

The sample included youth participating in MATCH (Monitoring Activities of Teenagers to Comprehend their Habits study), an ongoing longitudinal study investigating patterns of physical activity participation. A total of 806 children (51% of those eligible) age 10.3 ± 0.6 years were recruited in the first year of the study from grade 5 and 6 classes in 17 schools in New Brunswick, Canada. Other students from the same schools joined the study in year 2 (*n* = 39) and 4 (*n* = 92), for a total of 937 participants (55% female) over 4 years. Data were collected in self-report questionnaires every 4 months beginning in Fall 2011. For the current study, we used data collected over 6 years in the first 16 data collection cycles. All participants provided written informed assent and their parents provided written informed consent. A detailed study protocol is published elsewhere [29].

### Study variables

#### Mental health

Mental health was assessed using the Mental Health Continuum-Short Form (MHC-SF) [12], which comprises 14 items measuring emotional (3 items), social (5 items) and psychological (6 items) well-being. Participants rated how often they felt “this way” during the last month on a 6-point Likert scale ranging from 0 (*never)* to 5 *(all the time)*. A total score (range 0–70) was computed for overall well-being; higher scores indicate better mental health. Scores from the MHC-SF have been shown to be valid, reliable and sex-invariant in assessing positive mental health in a sample of Canadian adolescents and young adults [30]. Scores on all three subscales of the MHC-SF had high internal consistency in MATCH (Cronbach’s α 0.90 to 0.94), as did scores for the total scale (α = 0.97).

#### Physical activity participation

PA participation was measured as free-time engagement in PA (i.e., outside school gym/physical education class) in a 36-item PA and sport checklist (e.g. hockey, basketball, ice skating, kayaking, karate). This questionnaire is similar to other self-report questionnaires used at this age [31, 32]; it assesses the frequency (i.e., never, once a month or less, 2–3 times per month, once a week, 2–3 times per week, 3–4 times per week, almost every day) and with whom (i.e., alone, with friends, with parents/ siblings, or with an organized group, team) each activity was undertaken. There are no standard methods to define regular exposure to PA in the litterature. Based on previous work on sport profile categorization, authors suggest that regular engagement in a given sport over at least 8 months per year can be defined as year-round participation in that sport [33, 34]. Similarly, in the present study, participants were categorized as engaging in PA for a given year (yes, no) if they reported participating in at least one activity per week, for a minimum of two of the three cycles during the year. In previous work using MATCH data [35], minimum frequency of one time per week was compared to 2–3 times per week to define sport profile categorization, with no substantial differences in the findings. A variable was created to quantify whether participants reported participating in PA for a total of 0, 1, 2, 3, 4 or 5 years.

#### Autonomy

The 7-item autonomy subscale of the Basic Psychological Needs in Life Scale [36] was used to assess perceived autonomy. Items are scored on a 7-point Likert scale ranging from 1 (*not at all true*) to 7 (*very true*). The original scale was adapted to the physical activity context by including the word “physical activity” with following instructions: “*The following statements represent different feelings people have when they engage in physical activity. Using the scale provided, please answer the following questions by considering how you typically feel when participating in physical activity.”* An example of a revised item is: *“when I participate in physical activity, I feel like I can pretty much be myself”*. Based on psychometric assessment using the first 9 cycles of MATCH data [37], the three negatively worded items from the Basic Psychological Needs in Life Scale were removed. Similar to previous reports in studies of adolescents [19], the internal reliability of the score was good in the MATCH database (Cronbach’s α = 0.89).

#### Competence

Perceived competence was measured using the Intrinsic Motivation Inventory [38] which comprises six items scored on a 7-point Likert scale ranging from 1 (*not at all true*) to 7 (*very true*). The same adaptation and instructions as for the autonomy scale were provided. An example of a revised item is: *“I think I am pretty good at physical activity”*. Similar to the autonomy scale, the one negatively worded item in the Intrinsic Motivation Inventory scale was removed [39]. Similar to previous reports in adolescents [40], our findings support the internal reliability of the score (Cronbach’s α = 0.92).

#### Relatedness

The Relatedness to Others in Physical Activity Scale comprises 6 items (e.g., *“I feel like I am part of a group who share my goals”)* assessing perceived relatedness to others, with each item representing feelings people have when they engage in physical activity [41]. While the Relatedness to Others in Physical Activity Scale was developed to assess perceptions of relatedness in physical activity contexts in adults [41], validity and reliability of the scores have been demonstrated in adolescents [42]. In the present study, Cronbach’s α = 0.96.

#### MVPA

MVPA was assessed using a 2-item self-report measure developed specifically for adolescents [43]. Participants were asked to read the following statement: “*Physical activity is an activity that increases your heart rate and makes you get out of breath some of the time. Physical activity can be done in sports, playing with friends, or walking to school. Some examples of physical activity are running, brisk walking, rollerblading, biking, dancing, skateboarding, swimming, soccer, basketball, football, and surfing*” and then asked: “*Over the course of the week (past 7 days), how many days were you physically active for a total of at least 60 minutes per day?*” and “*Over the course of a typical or usual week, how many days are you physically active for a total of at least 60 minutes per day?*” Response options ranged from 0 to 7 days. The two items were averaged to estimate the number of days of self-report MVPA per week. Based on previous psychometric assessment, this measure is recommended to assess MVPA [44]; scores have moderate to high test-retest reliability (intra-class correlation = .77) and moderate correlation with accelerometer-measured MVPA (Pearson correlation = .40) in early adolescents have been reported [43].

#### Covariates

Mediation analysis based on the causal inference approach relies on the assumption of no unmeasured confounding [45]. Based on the literature, the most important confounders of the associations between PA and mental health, between PA and each potential mediator, and between the mediators and mental health, included sex, age and income [46, 47]. Previous mental health level could potentially confound the associations. However, no measure of mental health was available at baseline. Neighbourhood income was determined by matching 6-digit postal codes reported by participants, with the mean income of persons ≥15 years in 2011 in each participants’ neighbourhood, as proposed in the National Household Survey [48]. Participants were grouped into neighbourhood income groupings using tertiles.

### Analysis

Preliminary analyses comprised descriptive statistics to assess distributions, identify outliers and compute proportions, means and standard deviations. Mediation analyses were then performed to identify mechanisms on the causal pathway between PA and mental health, using the counterfactual definition of the total effect (TE) that allows decomposition into natural direct (NDE) and natural indirect (NIE) effects [45]. The NDE expresses how much mental health would change if the number of years participating in PA increased by 1 with the mediators set at the level they would have been in the absence of PA participation (i.e., the value of self-report MVPA, autonomy, competence and relatedness when the number of years of PA = 0). The NIE expresses how much the mental health score would change on average if the value of the mediators were changed from the level they would take when the number of years of PA = 0 to the level they would take if the number of years of PA = 1, while the number of years of PA is fixed at level 1*,* which is equivalent to suppressing the direct effect of PA. The mediated proportion of the association is expressed by the ratio NIE/TE [45]. As a first step, four mediation models were estimated (one for each mediator separately). Then, three joint mediation models with two mediators were estimated, including 1) perception of one basic psychological need (i.e., autonomy, competence, or relatedness) and 2) self-report MVPA as mediators. A joint mediation model including all four mediators (i.e., 4-mediator model) was estimated to distinguish the relative importance of each mediator in the same model. Pure natural indirect effects (PNIE), which represent the proportion of the association due to mediation only, specific to each mediator included in the joint models, were identified using natural effect decomposition [49]. Estimations were obtained in parametric models based on linear regressions. Exposure, mediators and the outcome were all modeled as continuous variables and the scores for mediator variables were standardized to facilitate interpretation of the results. To avoid reverse causation bias, we considered consecutive rather than concurrent measures: potential confounders (cycle 1), exposure (cycle 1 to 15), mediators (cycle 15) and outcome (cycle 16). The significance level was set at *p* < 0.05. Statistical analyses were performed with R Software Version 3.4.2. Mediation analyses were performed using the *medflex* package, which computes confidence intervals for TE, NDE and NIE estimates using the robust sandwich variance estimator for linear regression [50]. PNIE were calculated [49] and confidence intervals were estimated by nonparametric bootstrap resampling.

Missing values for variables included in the analytical model ranged from 0 to 25.2%; 189 participants (45%) had complete data on all variables. We used multiple imputation by chained equations with 10 imputation sets using the *mice* package [51]. Imputation was conducted for 424 participants not lost to follow-up at cycle 16. Imputation models included all variables considered in the mediation models, as well as baseline self-report MVPA.

## Results

The analytical sample comprised 424 participants (57% female, mean (SD) age at baseline = 10.2 (0.6) years). Characteristics of participants retained for analysis and missing values for each variable of interest are presented in Table 1.

**Table 1:** Characteristics of participants retained for analysis and missing data (*n* = 424)

	Time of measure (cycle)	Range	n (%) or mean (SD)^a^	Missing data *n* (%)
Sex (female), *n* (%)	1	–	242 (57.1)	0
Age (y) at inception, mean (SD)	1	10–11	10.2 (0.6)	0
Neibourhood income tertile, *n* (%)	1		**–**	71 (16.7)
High	–	–	155 (43.9)	–
Moderate	–	–	98 (27.8)	–
Low	–	–	100 (28.3)	–
PA participation (# year), *n* (%)	1–15	1–7	–	140 (33.0)
0	–	–	3 (10.6)	–
1	–	–	10 (3.5)	–
2	–	–	16 (5.6)	–
3	–	–	22 (7.8)	–
4	–	–	64 (22.5)	–
5	–	–	169 (59.5)	–
Autonomy, mean (SD)	15	1–7	4.8 (1.6)	103 (24.3)
Competence, mean (SD)	15	1–7	5.0 (1.5)	99 (23.3)
Relatedness, mean (SD)	15	1–6	4.6 (1.4)	107 (25.2)
MVPA, # active days/wk., mean (SD)	15	0–7	4.6 (2.0)	96 (22.6)
Mental health, mean (SD)	16	0–70	48.4 (17.2)	30 (7.1)

^a^% and mean(SD) among valid data, excluding missing data

*MVPA* Moderate-to-vigorous physical activity

No meaningful differences in sex (*p* = .30) or age (*p* = .19) were observed between participants retained (*n* = 424) and not retained (*n* = 513). Participants included were more likely to have household incomes at baseline in the highest tertile grouping (44% vs. 26%, *p* < .01). We explored bivariate correlations and found that mental health was moderately correlated with each of perceived autonomy, competence and relatedness (Pearson’s r ranged from .33 to .50, all *p* < .05); perceived autonomy, competence and relatedness were moderately inter-correlated (Pearson’s r ranged from .64 to .76, all *p* < .05), suggesting that they capture distinct, but related, concepts.

Because the interactions between the exposure and each mediator were not statistically significant in linear regression analyses assessing TE (results not shown), all interaction terms were dropped from the mediation analysis [52]. Table 2 presents NDE and NIE estimates for the mediated effects of each of autonomy, competence, relatedness and self-report MVPA (assessed in separate models). We observed statistically significant estimates for NIE for all four variables. The NIEs suggest that a one-standard deviation increase in autonomy, competence, relatedness and self-report MVPA was associated with an increase of 1.64 (95%CI = 0.73, 2.55), 1.35 (95%CI = 0.39, 2.31), 2.14 (95%CI = 1.06, 3.22) and 1.77 (95%CI = 0.75, 2.79) units in the mental health score, respectively, when number of years of PA is fixed. Computation of the mediated proportion suggests that perceived autonomy, competence and relatedness and self-report MVPA mediate approximately 60, 50, 79 and 66% of the association, respectively.

**Table 2:** Beta coefficients and 95% Confidence Intervals (CI) of the natural direct, natural indirect and total effects of number of years in PA on mental health in single mediator models considering mediation through autonomy, competence, relatedness and MVPA (imputed data, *n* = 424)

	Autonomy		Competence		Relatedness		MVPA	
	$\hat{\upbeta}$	95% CI	$\hat{\upbeta}$	95% CI	$\hat{\upbeta}$	95% CI	$\hat{\upbeta}$	95% CI
NDE	1.06	(−1.08, 3.20)	1.35	(−0.99, 3.68)	0.56	(−1.69, 2.81)	0.93	(−1.40, 3.25)
NIE	**1.64**	**(0.73, 2.55)**	**1.35**	**(0.39, 2.31)**	**2.14**	**(1.06, 3.22)**	**1.77**	**(0.75, 2.79)**
TE	**2.70**	**(0.69, 4.70)**	**2.70**	**(0.69, 4.70)**	**2.70**	**(0.69, 4.70)**	**2.70**	**(0.69, 4.70)**

^a^Models include only one mediator (Autonomy; Competence; Relatedness; MVPA)

*NDE* Natural Direct Effect, *NIE* Natural Indirect Effect, *TE* Total effect, *CI* Confidence Intervals

Bold indicates statistically significant results *p* < .05

In joint mediation models including both one basic psychological need and self-report MVPA as mediators (Table 3), the proportion of the association mediated by perceived autonomy, competence and relatedness was 71, 27, and 51%, respectively. The proportions mediated by self-report MVPA in the autonomy and competence models were approximately 27 and 31%, respectively. The pure natural indirect effect (PNIE) of self-report MVPA in the relatedness model was not statistically significant. In the 4-mediator model, relatedness mediated the largest proportion of the association, followed by autonomy and self-report MVPA with similar PNIE estimates (Table 4). The proportion mediated should be interpreted cautiously when the NDE and NIE are in opposite directions, which explain why the NIE is larger than the TE; beta coefficient estimates might be more useful to compare the contribution of each mediator in these models.

**Table 3:** Beta coefficients and 95% Confidence Intervals (CI) for the natural direct, natural indirect and total effect of number of years in PA on mental health in joint mediation models including MVPA and each of autonomy, competence and relatedness (imputed data, *n* = 424)

	Autonomy _(M1)_		Competence _(M1)_		Relatedness _(M1)_	
	+ MVPA		+ MVPA		+ MVPA	
	$\hat{\upbeta}$	95% CI	$\hat{\upbeta}$	95% CI	$\hat{\upbeta}$	95% CI
NDE	0.10	(−2.31, 2.59)	0.38	(−2.09, 2.86)	−0.26	(−2.87, 2.34)
NIE_M1 + MVPA_	**2.60**	**(1.39, 3.80)**	**2.31**	**(1.13, 3.50)**	**2.96**	**(1.54, 4.38)**
PNIE_M1_	**1.47**	**(0.60, 2.33)**	**1.07**	**(0.19, 1.95)**	**1.93**	**(0.93, 2.94)**
PNIE_MVPA_	**1.13**	**(0.11, 2.15)**	**1.25**	**(0.19, 2.30)**	1.03	(−0.06, 2.12)
TE	**2.70**	**(0.69, 4.70)**	**2.70**	**(0.69, 4.70)**	**2.70**	**(0.69, 4.70)**

^a^Models include two mediators (MVPA + Autonomy; MVPA + Competence; MVPA + Relatedness)

*NDE* Natural Direct Effect, *NIE* Natural Indirect Effect, *TE* Total effect, *CI* Confidence Intervals

Bold indicates statistically significant results *p* < .05

**Table 4:** Beta coefficients and 95% Confidence Intervals (CI) for the natural direct, natural indirect and total effect of number of years in PA on mental health in joint mediation model including four mediators (imputed data, *n* = 424)

	4-Mediator Model^a^	
	$\hat{\upbeta}$	95% CI
NDE	−0.22	(−2.73, 2.29)
NIE_TOTAL_	**2.92**	**(1.52, 4.31)**
PNIE_AUTONOMY_	**1.06**	**(0.25, 1.88)**
PNIE_COMPETENCE_	−0.67	(−1.43, 0.09)
PNIE_RELATEDNESS_	**1.45**	**(0.42, 2.48)**
PNIE_MVPA_	**1.07**	**(0.10, 2.05)**
TE	**2.70**	**(0.69, 4.70)**

^a^Model include four mediators (Autonomy + Competence + Relatedness + MVPA)

*NDE* Natural Direct Effect, *NIE* Natural Indirect Effect, *TE* Total effect, *PNIE* Pure Natural Indirect Effect, *CI* Confidence Intervals

Bold indicates statistically significant results *p* < .05

## Discussion

Previous research supports that PA leads to benefits in mental health and a reduced risk of mental disorders [46, 53]. However, our study is the first to investigate psychosocial factors and MVPA as mechanisms underpinning the positive effect of PA on mental health in adolescents. Identification of the mediating role of autonomy, competence, relatedness and MVPA provides a unique contribution to the literature by demonstrating that the positive effect of PA in childhood and early-to-mid adolescence on later mental health is attributable, at least in part, to these mechanisms.

Several mechanisms have been proposed to explain the effect of PA on mental health and mental disorders. Although we did not assess specifically biological mechanisms, the benefits of PA (i.e., energy expenditure through movement [54]), and especially MVPA, on mental health may relate to the secretion of serotonin [55] and endorphin [56], known respectively for their antidepressant and analgesic effects. PA also impact cortisol regulation [57], which reduces physiological reactivity to stress. However, these biological mechanisms may not be exhaustive in explaining the positive effect of PA participation on mental health. Alternative or additional mechanisms may be that PA participation enhances psychosocial determinants of mental health including self-esteem [4] and development of a wide and supportive social network [9]. The experience of PA participation, also favors the satisfaction of feelings of autonomy, competence and relatedness specific to the PA context [39]. Correspondingly, we found that more years of PA participation is associated with higher autonomy, competence and relatedness perceptions, which in turn positively impact mental health in late adolescence. In joint mediation models assessing the mediating effect of each basic psychological need conjointly with self-report MVPA, the PNIE suggests that each of autonomy, competence and relatedness explain a larger proportion of the association than MVPA in the 2-mediator models. The 4-mediator model reinforce those results.

Most MATCH participants were exposed to social PA contexts; less than 10% (from 2.8% in year 2 to 9.4% in year 4, see Additional file 1) engaged in individual sport only. Thus, the indirect effect of the PA and mental health association through perceived autonomy and competence could relate to the high quality of social interactions experienced by youth in PA. Ryan and Deci [13] contend that satisfaction of psychological needs does not happen in isolation, but in high quality social experiences. These findings align with research suggesting that PA behavior (i.e., cooperation, supporting each other’s success) fosters feelings of autonomy and competence [23, 24]. The indirect effect through perceived relatedness in our study is consistent with past work suggesting that PA in social contexts (e.g. team sport) can enhance perceived social acceptance [58], and may provide adolescents with opportunities to bond with their peers and feel connected, all of which can influence mental health positively [59]. Our findings also align with the Positive Youth Development model which posits that when PA promotes positive psychological and social environments through supportive relationships or positive social norms, greater benefits on mental health are observed [1]. Some studies suggest greater benefits for mental health from participation in team activities [46, 60, 61], although others [53, 62, 63] suggest no added value for team versus individual sport. Future studies should investigate the mediating effect of autonomy, competence, relatedness and MVPA on the association between a PA variable comparing individual vs. group/team contexts and mental health.

### Strengths and limitations

Strengths of this study include its longitudinal design including data collection every 4 months over 6 years from childhood into adolescence. We used measures of psychological needs that have been validated in a sport context. Further, this study used a valid comprehensive measure of mental health that included emotional, psychological and social dimensions of well-being, and thus provided a global mental health assessment, that has been found to predict the risk of common mental disorders [11].

Limitations include that self-report PA measures are subject to misclassification. Baseline mental health level in late childhood could confound the association of interest such that participants with better mental health may have been more likely to participate in PA, which could (partially) explain the positive association between consistent PA participation during the first 5 years of the study and mental health in year 6. In MATCH, mental health was measured for the first time in cycle 16, precluding adjustment for baseline mental health. MATCH was originally designed to describe PA and sport participation during childhood and adolescence. Thus, none of well-being, quality of life or mental health were assessed at baseline. Future studies should adjust for potential confounding by past mental health. However, a recent study using Mendelian randomization and accelerometer-assessed PA supports the hypothesis that PA protects against depression, but found no evidence that depression influences PA negatively [64]. Residual confounding might have been an issue; future studies should consider adjusting for chronic physical illness during childhood, living in urban vs. rural areas, family social environment and parents’ mental health. Previous studies suggest that PA and physical self-concept, a hierarchical construct comprising related subcomponents such as strength, body fat, endurance/fitness, sports competence, coordination, health, appearance and flexibility [65] are reciprocally related [66] and that physical self-concept mediates the association between PA and sport participation and depressive symptoms among girls in late adolescence [67]. Future studies should consider investigating whether self-concept is a mediator of the association between PA and mental health. The mediation models were estimated without interaction between exposure and mediator or between mediators (in joint mediation models). In multivariate linear regression models, we tested interaction between the exposure and each mediator and the interaction between mediators and none of the interactions were statistically significant. This could represent a real absence of interaction or may be attributable to the small sample size (which may render the analysis underpowered to detect interaction). Future studies using larger sample sizes should include statistically significant interactions, if any, in the mediation models. Selection bias due to loss-to-follow-up may have been an issue. Therefore, our results need replication in other settings. Finally, use of a purposive sample may limit generalizability of the results.

## Conclusion

This study suggests that level of autonomy, competence and relatedness experienced in PA contexts as well as MVPA explains the association between PA participation and mental, health at least in part. Our results support the development of strategies to encourage youth to engage and remain involved in PA contexts that enhance perceptions of autonomy, competence and relatedness, in addition to strategies that promote increased MVPA, to promote mental health in adolescents.

## Supplementary information

**Additional file 1: Table S1.** Physical activity/sport participation status according to context in each of the first 5 years of the study among participants in cycle 16 (*n* = 424).

## Data Availability

The original data analyzed for these analyses are available through a data sharing agreement with the MATCH study research team. More information on this may be obtained from the principal investigator of the MATCH study, Dr. Mathieu Belanger.

## Abbreviations

MATCH: Monitoring Activities of Teenagers to Comprehend their Habits
MVPA: Moderate-to-vigorous Physical Activity
NDE: Natural Direct Effect
NIE: Natural Indirect Effect
PNIE: Pure Natural Indirect Effect
TE: Total Effect
