# Supplementary material for: Mechanisms underpinning the association between physical activity and mental health in adolescence: a 6-year study
Source: Int J Behav Nutr Phys Act. 2020 Jan 31;17:9. doi: 10.1186/s12966-020-0911-5 (PMC6993479; doi:10.1186/s12966-020-0911-5)
Supplement: Supplementary file 1 — Additional file 1: Table S1. Physical activity/sport participation status according to context in each of the first 5 years of the study among participants in cycle 16 (n = 424). [file 12966_2020_911_MOESM1_ESM.docx]

Supplementary material

**Table S1. Physical activity/sport participation status according to context in each of the first 5 years of the study among participants in cycle 16 (*n* = 424)**

|  | Year 1 | | Year 2 | | Year 3 | | Year 4 | | Year 5 | |
| --- | --- | --- | --- | --- | --- | --- | --- | --- | --- | --- |
|  | n | % | n | % | n | % | n | % | n | % |
| **Physical activity context** |  |  |  |  |  |  |  |  |  |  |
| 1. No PA | 30 | 8.8 | 43 | 11.9 | 57 | 15.8 | 74 | 19.2 | 76 | 19.3 |
| 1. IND only | 10 | 2.9 | 10 | 2.8 | 22 | 6.1 | 36 | 9.4 | 34 | 8.6 |
| 1. UNORG only | 41 | 12.0 | 40 | 11.1 | 26 | 7.2 | 30 | 7.8 | 22 | 5.6 |
| 1. ORG only | 26 | 7.6 | 36 | 10.0 | 42 | 11.6 | 52 | 13.5 | 64 | 16.2 |
| 1. IND + UNORG | 37 | 10.8 | 43 | 11.9 | 38 | 10.5 | 36 | 9.4 | 36 | 9.1 |
| 1. IND + ORG | 11 | 3.2 | 15 | 4.2 | 31 | 8.6 | 44 | 11.4 | 58 | 14.7 |
| 1. UNORG + ORG | 77 | 22.5 | 69 | 19.2 | 58 | 16.1 | 40 | 10.4 | 32 | 8.1 |
| 1. IND + UNORG + ORG | 110 | 32.2 | 104 | 28.9 | 87 | 24.1 | 73 | 19.0 | 72 | 18.3 |
| Total | 342 | 100.0 | 360 | 100.0 | 361 | 100.0 | 385 | 100.0 | 394 | 100.0 |

PA = Physical activity, IND = Individual (alone), UNORG = Unorganized (with parents/siblings), ORG = Organized (with an organized group or team).
